# ZNF671 Silencing Affects Signaling Pathways in Head and Neck Cancer via Activation of Oncogenic Non-Coding RNAs

**DOI:** 10.3390/biomedicines12112482

**Published:** 2024-10-29

**Authors:** Kendra Smith, Rufa’i Umar Zubair, Richard V. Smith, Stelby Augustine, Nicholas F. Schlecht, Thomas J. Ow, Michael B. Prystowsky, Thomas J. Belbin

**Affiliations:** 1 Division of Biomedical Sciences, Faculty of Medicine, Memorial University of Newfoundland, St. John’s, NL A1B 3V6, Canadaruzubair@mun.ca (R.U.Z.); 2 Discipline of Oncology, Faculty of Medicine, Memorial University of Newfoundland, St. John’s, NL A1B 3V6, Canada; 3 Department of Otorhinolaryngology—Head and Neck Surgery, Montefiore Med Center, 3400 Bainbridge Ave., Bronx, NY 10467, USA; 4 Department of Pathology, Albert Einstein College of Medicine, 1300 Morris Park Ave., Bronx, NY 10461, USA; michael.prystowsky@einsteinmed.edu; 5 Department of Cancer Prevention and Control, Roswell Park Comprehensive Cancer Center, Elm and Carlton Streets, Buffalo, NY 14263, USA

**Keywords:** ZNF671, LINC00665, oral cavity, squamous, carcinoma

## Abstract

Background: Novel ZNF genes, such as ZNF671, that are located on chromosome 19q13 are known to be hypermethylated at a high frequency in HNSCC as well as in other epithelial solid tumors. Their function is largely unknown. Results: Here, we show that ZNF671 is epigenetically silenced in HNSCC primary tumors compared to matched adjacent normal tissue. Moreover, low expression of ZNF671 is significantly associated with decreased survival in HNSCC patients. Over-expression of ZNF671 in UM-SCC-1 oral cancer cells resulted in a significant reduction in tumor cell mobility and invasion compared to the empty-vector control cells. Transcriptomic analysis showed that ZNF671 re-expression resulted in a significant decrease in the expression of a major oncogenic long non-coding RNA LINC00665. Conclusions: Together, these results suggest that epigenetic silencing of ZNF671 may activate multiple oncogenic signaling pathways via the resulting up-regulation of LINC00665.

## 1. Introduction

Head and neck squamous cell carcinomas (HNSCCs) still rank among the most common tobacco and alcohol associated malignancies in men and women worldwide [1]. The 5-year survival rates have only modestly improved over the last decade and remain at around 50%. In addition, recurrence of the disease following treatment is observed in about 50% of patients with high rates of associated mortality. In Canada, more than 4300 people will develop cancer of the head and neck this year; over 1600 of them will die from this disease [2].

In HNSCC, promoter methylation of tumor suppressor genes appears to be a common mechanism of transcriptional silencing [3]. The identification of novel genes specifically affected by DNA methylation represents a way forward to the identification of genes with relevance as potential clinical biomarkers, and in identifying potential new tumor suppressor genes and understanding their mechanisms of action. Our group and others have previously completed genome-wide scans of aberrant DNA methylation in DNA samples from HNSCC primary tumors [4,5,6]. Our previous study identified a cluster of epigenetically-silenced Krüppel-type zinc finger protein (ZNF) genes located on chromosome 19q13 in all anatomic sub-sites of HNSCC [5]. One of these was ZNF671, a novel Krüppel-type zinc finger protein that is epigenetically-silenced with high frequency in HNSCC cases.

Here, we examine the expression of ZNF671 and its aberrant promoter DNA methylation in an independent patient cohort of the Cancer Genome Atlas (TCGA). We examine the effects of overexpression of ZNF671 in HNSCC cell line UM-SCC-1. Finally, we shed some light on long non-coding RNA LINC00665 as a novel target for ZNF671, and its subsequent effect on multiple cancer-related signaling pathways.

## 2. Materials and Methods

### 2.1. Analysis of Global Gene Expression and DNA Methylation Data from the Cancer Genome Atlas (TCGA) and Einstein/Montefiore Patient Cohorts

Global gene expression, DNA methylation, clinical characteristics, and overall survival data, current as of December 2020, for 516 primary HNSCC patients were downloaded from the TCGA database [7]. These represented data from three major anatomic site: oral cavity (alveolar ridge, buccal mucosa, floor of mouth, hard palate, and oral tongue), oropharynx (base of tongue, uvula, soft palate, and tonsil), and larynx (hypopharynx, and larynx). DNA methylation data were measured using Illumina HumanMethylation450k bead-chip(San Diego, California). Beta-values were converted to M-values using the log transformation, as described previously [8]. For downstream analysis, the M-value was more statistically valid for the differential analysis of methylation levels [8]. Gene expression was represented as normalized gene counts from RNA seq data. Overall survival was measured as the time in days between the date of surgery and the date of death or the last follow-up. Einstein cohort clinical data and ZNF671 expression data, measured using the Illumina HT-12 DirectHyb expression bead-chip (Illumina, San Diego, California) were downloaded from the Albert Einstein College of Medicine Head and Neck Cancer Database [9]. In this cohort, survival is represented as end death of disease.

Summary gene expression and DNA methylation data for ZNF671 are presented as Mean ± SD. We compared ZNF671 gene expression and DNA methylation between HNSCC primary tumor and adjacent non-tumor tissue samples using paired *t*-test, and all were graphed using the *boxplot* function in R (version 4.3.1). Survival data for both the TCGA and Einstein Montefiore patient cohorts were assessed using the log-rank statistical test to assess differences between survival curves following patient stratification based on median ZNF671 tumor expression. All analysis was completed using the *survival* package 3.4-0 in R and plotted using *survminer* 0.5.2 [10]. In all cases, a threshold *p*-value of *p* < 0.05 was accepted as statistically significant.

### 2.2. Lentiviral Transduction of HNSCC Cell Lines

Overexpression of ZNF671 protein in oral squamous cell carcinoma cell line UM-SCC-1 was carried out by lentiviral transduction as described previously, and according to the recommendations of the manufacturer [11]. UM-SCC-1 is an oral cavity squamous cell carcinoma cell line isolated from a tumor located on the floor of the mouth of a 73-year-old male patient. The original tissue was described as a T2N0M0 moderately differentiated local recurrence. This cell line is negative for Human Papillomavirus 16 (HPV-16). In order to stably overexpress ZNF671 in UM-SCC-1 cells, we utilized the pLenti-C-Myc-DDK-P2A-Puro construct vector expressing the ZNF671 gene as a Flag-tagged fusion protein under the control of the CMV promoter (Cat#RC206413L3, Origene, Rockville, MD, USA). We utilized the empty pLenti-C-Myc-DDK-P2A-Puro vector as a negative control (Cat# PS100092, Origene). Total RNA isolation from UM-SCC-1 cells was carried out by the RNeasy total RNA kit (Cat# 74104, Qiagen, Hilden, Germany). We confirmed expression of the ZNF671 fusion transcript by Taqman real-time PCR (ZNF671: Hs01087685_m1) (Thermo Fisher Scientific, Waltham, MA, USA).

### 2.3. Measurement of ZNF671 Protein Expression

Levels of ZNF671 fusion protein in transduced cells were confirmed as described previously for ZNF proteins [11]. Protein measurement for tagged-ZNF671 was carried out by Western blot using total cellular protein (40 µg). These were resolved on a 10% SDS-PAGE gel and transferred onto a nitrocellulose membrane (Cat# 1620115, BioRad, Hercules, CA, USA). Tagged ZNF671 protein was detected using a Flag primary antibody (1:2000) (Cat# TA50011, Origene) and secondary antibody (goat anti-mouse HRP (1:5000) (Cat# 115-035-071, Jackson Immunoresearch, West Grove, PA, USA). Primary HRP signal was measured using Amersham ECL Select Western Blotting Detection Reagent (RPN2235). All images were analyzed on a Biorad Chemidoc MP Imaging System using Bio-rad Laboratories Image Lab software (version 6.1). All membranes were re-probed for β-actin (1:2000) (Cat# MABT523, Millipore Sigma, Darmstadt, Germany) in the presence of 0.05% sodium azide as a loading control. In the case of actin, the secondary antibody was goat anti-rabbit HRP (1:10,000) (Cat# 65-6120, Invitrogen, Waltham, MA, USA). HRP signal was detected using SuperSignal West Pico PLUS Chemiluminescent Substrate (Cat# 34579, Thermo Fisher Scientific).

### 2.4. Measurement of Tumor Cell Growth, Migration and Invasion

Viable cell counts were measured every 48 h by Trypan Blue exclusion. These were reported as mean count ± SD in triplicate measurements. Differences between cell counts were assessed by single factor. UM-SCC-1 cell migration properties were measured using the Radius 96-well cell migration assay (Cat# CBA-126, Cell Biolabs, San Diego, CA, USA). Cell-free area captured at 0 and 48 h post gel removal was measured using Adobe Photoshop (version 23.2.2.325).

Tumor cell invasion assays were performed using BioCoat Matrigel Invasion Chambers (Cat# 08-774-122, Thermo Fischer Scientific), as described previously [12]. Briefly, invasion chambers were first hydrated with complete media for 2 h at 37 °C, 5% CO_2_. Cells were harvested with Accutase and approximately 1.0 × 10^5^ cells were suspended in 0.5 mL serum free media (0.7% BSA) and added to the top well of the invasion chamber with the bottom well containing 0.1 nM mouse EGF (Cat # SRP3196, Sigma Aldrich) diluted in serum free media (0.7% BSA). Chambers were incubated for 24 h at 37 °C, 5% CO_2_. Cells were then fixed with formalin for 15 min and stained with Crystal Violet staining solution (0.2% crystal violet, 2% ethanol in dH_2_O) for 10 min. Non-invading cells were removed from the membrane with a moistened cotton swab. Membranes were cut from the chamber, mounted on a microscope slide and imaged with a flatbed scanner. The area of the membrane covered with invading cells was quantified using ImageJ software (version 1.54, National Institute of Health, Bethesda, Maryland USA).

### 2.5. Whole Transcriptomic Analysis of Gene Expression

Whole transcriptome analysis of gene expression differences in ZNF671 overexpressing UM-SCC-1 cells was carried out by RNA sequencing on an Illumina NovaSeq 6000 S4 PE100 (Genome Quebec, Montreal, QC, USA) as paired 100 bp reads. Resulting reads were assessed for quality using FastQC (version 0.11.9) [13]. The resulting fastq read files were aligned to the Human hg38 reference genome using RNA-Star aligner (Galaxy Version 2.7.8a) with default settings. Transcripts for a given gene were counted using *featureCounts* (Galaxy version 2.0.1) [14,15]. Identification of differentially expressed genes when comparing ZNF671 overexpressing and empty vector control UM-SCC-1 cells was carried out using the negative binomial distribution with *DeSeq2* (Galaxy Version 2.11.40.7), with a Benjamini—Hochberg adjusted *p*-value of less than 0.05 [16].

Identification of overrepresented groups of genes was carried out using *GOseq* (Galaxy Version 1.44.0) [17]. *GOseq* was used for gene set enrichment analysis with RNA seq data, as it could account for gene length bias in the detection of over-represented genes ([17]). *GOseq* was used to calculate a probability weighting function (PWF), which gave us the probability that a gene would be differentially expressed (DE) based on its length alone. This function was then used to weigh up the chance of selecting each gene when forming a null distribution for gene ontology (GO) category membership. Random sampling was then utilized in order to generate a null distribution for GO category membership, and to calculate each category’s significance for over representation amongst the differentially expressed genes. Gene ontology categories tested included Molecular Function (GO:MF), Cellular Component (GO:CC), and Biological Process (GO:BP). The distribution of differentially expressed genes in a given category was assessed using a Wallenius non-central hypergeometric distribution [17]. The over representation of a given GO term among these differentially expressed genes was assessed by *p*-value while adjusting for multiple testing using Benjamini—Hochberg correction [18].

### 2.6. Knockdown of ZNF671 Gene Expression by siRNA Transient Transfection in Human Epithelial Keratinocytes

Human epithelial keratinocytes (Hekn) were purchased from ATCC (Manassas, Virginia, US) (PCS-200-010) and maintained at 37 °C, 5%CO_2_. Cells were cultured in Dermal Cell Basal Medium (ATCC PCS-200-030) supplemented with Keratinocyte Growth Kit (ATCC PCS-200-040). Knockdown of ZNF671 gene expression by siRNA was carried out essentially as described previously [19]. ZNF671 siRNA oligos used were as follows: siGENOME siZNF671: Cat. D-014473, Target sequences: 5′-GGACAGAGGUAUCACAGGU-3′, 5′-GGAGAGAAUUCAUCCGGAA-3′, 5′-CCUUACACCUGGCUAAAUA-3′, 5′-GAUUAUGAGUGUAGCAGAU-3′. Accell Green Non-targeting siRNA: Cat. D-001950, Target sequence: 5′-UGGUUUACAUGUCGACUAA-3′ (Horizon Discovery, Lafayette, Colorado, USA). Cells were then incubated for 48 h without changing medium. Knockdown of ZNF671 gene expression was confirmed by Taqman real-time PCR using the protocol as described by the manufacturer (ThermoFisher Scientific, Waltham, MA, USA).

## 3. Results

### 3.1. Epigenetic Silencing of ZNF671 in HNSCC Primary Tumors Has Prognostic Relevance for This Disease

**Figure 1:**
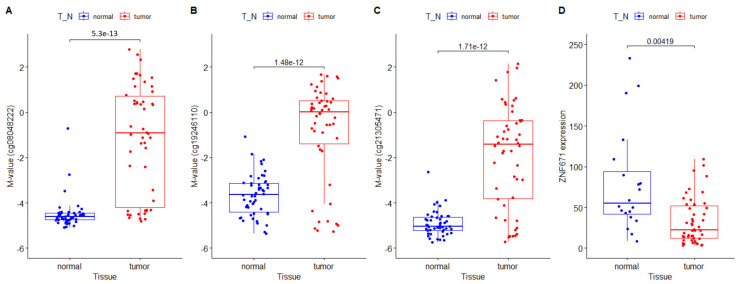
(**A**–**C**) Comparison of DNA methylation measurements (M-values) between primary tumor (red) and matched adjacent non-tumor tissue (blue) for 50 HNSCC patients from the TCGA. Specific promoter CpG loci for ZNF671 (cg08048222, cg19246110 and cg21305471) are shown on the y-axis label. (**D**) Comparison of ZNF671 RNA transcript levels in tumor and matched non-tumor tissues as measured by RNA seq data from 30 TCGA patients. Each graph includes a *p*-value for *t*-test comparisons between each matched group of samples.

The ZNF671 gene, located on chromosome 19q13, codes for a protein of 534 amino acids (61 kDa). The protein is categorized as a KRAB-ZNF protein containing ten C_2_H_2_ zinc finger domains as well as an N-terminal KRAB domain. Our first objective was to validate our previously reported findings that ZNF671 was epigenetically silenced in HNSCC [5]. We first compared measurements of DNA methylation, expressed as M-values, between primary tumor and matched adjacent non-tumor tissue, for 50 HNSCC patients from the TCGA. These measurements of DNA methylation (M-values) for all three CpG loci located within the ZNF671 promoter showed significantly increased DNA methylation in the primary tumor DNA compared with matching non-tumor tissue DNA from the same patient (cg08048222: −1.17 ± 2.43 (tumor) versus −4.49 ± 0.66 (non-tumor) *p* < 0.001, cg19246110: −0.80 ± 2.12 (tumor) versus −3.65 ± 0.92 (non-tumor) *p* < 0.001), and cg21305471: −1.92 ± 2.27 (tumor) versus −4.91 ± 0.55 (non-tumor) *p* < 0.001) (Figure 1A–C). Next we compared ZNF671 RNA transcript levels between primary tumor and matched adjacent non-tumor tissue for 30 of the same HNSCC patients using RNA sequencing data obtained from the TCGA. These data confirmed that ZNF671 expression was significantly down-regulated in HNSCC tumors compared with matching non-tumor tissue from the same patient (Figure 1D, *p* < 0.05). Taken together, the results confirm our initial findings of epigenetic downregulation of ZNF671 in a separate cohort of HNSCC patients.

**Figure 2:**
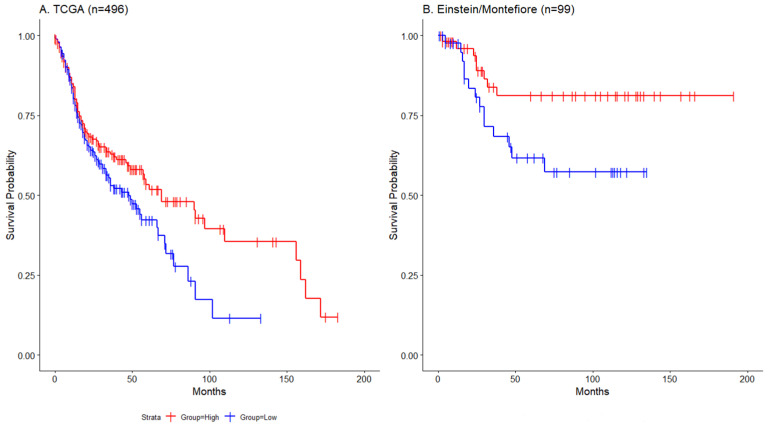
(**A**) Kaplan—Meier plot of overall survival (OS) for 496 TCGA HNSCC patients stratified by median ZNF671 expression as measured by RNA-seq data. (**B**) Kaplan—Meier plot of disease-specific survival for 99 Einstein HNSCC patients stratified by median ZNF671 expression. The blue line indicates patients with low tumor ZNF671 expression; the red line indicates high ZNF671 expression. Difference in survival between patient groups were assessed by Log-rank statistics.

Not all HNSCC tumors showed epigenetic silencing of ZNF671 expression. We therefore tested whether ZNF671 expression might have a negative correlation with promoter DNA methylation, and whether this expression might have prognostic significance in this disease. Utilizing the cohort of TCGA HNSCC patients, a scatter plot of DNA methylation versus gene expression for ZNF671 showed a significant negative correlation (Spearman correlation −0.58; Pearson correlation −0.52; Supplementary Figure S1). Moreover, survival analysis stratifying patients based on the median ZNF671 tumor expression demonstrated that patients whose primary tumors had low ZNF671 expression (less than the median expression) showed a significantly worse overall survival when compared to those with higher ZNF671 expression (Figure 2A, Log-rank, *p* < 0.05). This association was also validated in a separate cohort of 99 HNSCC patients’ data obtained from the Albert Einstein College of Medicine Head and Neck Cancer Database. In that cohort, patients whose primary tumors had low ZNF671 expression (less than the median expression) also showed a significantly worse disease-specific survival when compared to those with higher ZNF671 expression (Figure 2B, Log-rank, *p* < 0.05). Together, these observations support both an epigenetic mechanism for ZNF671 expression, and a possible role for this protein in head and neck carcinogenesis.

### 3.2. Over-Expression of ZNF671 Decreased Tumor Cell Mobility and Invasion

**Figure 3:**
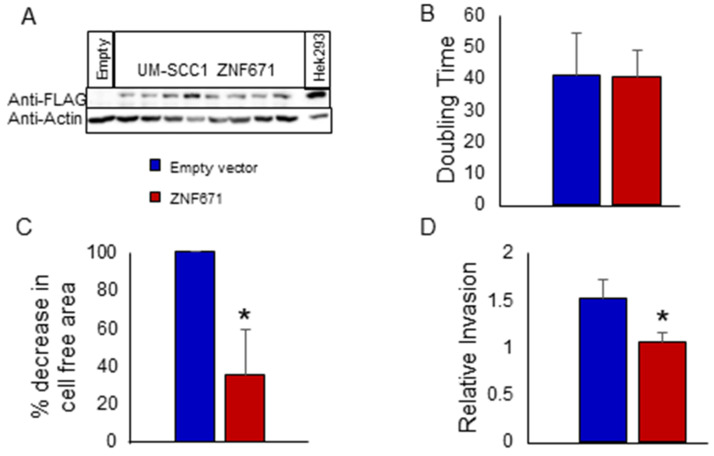
Overexpression of Flag-tagged ZNF671 in UM-SCC-1 oral cancer cells. (**A**) Western blot of total protein from eight individually transduced UM-SCC-1 clones was used to confirm overexpression of a Flag-tagged ZNF671 protein. Total protein from HEK-293 cells overexpressing the same Flag-tagged ZNF671 protein (right), and UM-SCC-1 transduced with the empty lentiviral vector (left) were used as positive and negative controls, respectively. Antibody Anti-actin was used as a loading control. Clones 3 and 4 were used in subsequent experiments. (**B**) Doubling time (h) of UM-SCC-1 cells overexpressing ZNF671 protein (red) compared to empty vector control cells (blue). (**C**) Migration assay comparing percentage decrease in cell-free area after 48 h in UM-SCC-1 cells overexpressing Flag-tagged ZNF671 (red) and empty vector UM-SCC-1 cells (blue). Corresponding images are shown as Supplementary Figure S2 (**D**). Relative invasion as measured by trans-well invasion assay comparing EGF chemoattractant versus non-attractant for each cell line at 48 h. Statistically significant differences between ZNF671 overexpressing cells and empty vector control cells are indicated by an asterisk (single factor ANOVA, *p* < 0.05).

In order to investigate potential tumor suppressive properties of ZNF671 in HNSCC cells, we overexpressed ZNF671 in the oral cancer cell line UM-SCC-1 by lentiviral transduction. As shown in Figure 3A, Western blot analysis showed an over-expression of Flag-tagged ZNF671 protein in eight UM-SCC-1 clones, with a molecular weight of 61 kDa (lanes 2–9). This protein was absent from the empty vector control cells (lane 1). Similarly, quantitation of ZNF671 RNA transcripts by Taqman quantitative real-time PCR (qPCR) revealed a significant increase in transcript abundance in the ZNF671 overexpressing cells compared to the empty vector controls. To date, attempts to overexpress ZNF671 in HNSCC cell lines SCC-15 and SCC-25 have been unsuccessful, or have resulted in the production of truncated proteins due to an unknown mechanism.

Overexpressing UM-SCC-1 oral cancer SCC cells were tested for changes in tumor cell phenotype. First, growth of UM-SCC-1 cells expressing the ZNF671 protein did not result in any significant increase in UM-SCC-1 doubling time (40.3 h) when compared to the empty vector control cells (40.7 h) (Figure 3B). However, migration of UM-SCC-1 cells, measured using the Radius 96-well cell migration assay, showed that expression of ZNF671 resulted in a significant decrease in tumor cell migration when compared to the empty vector control cells (Figure 3C). At 48 h, UM-SCC-1 cells overexpressing ZNF671 showed only a 36% decrease in cell-free area, compared with 100% for the empty vector control cells. Representative images of the cell migration assay are shown in Supplementary Figure S2. Similarly, overexpression of ZNF671 resulted in a significant decrease in tumor cell invasion compared to the empty vector control cells as measured by trans-well invasion assay (Figure 3D). From these results, we concluded that, when expressed in UM-SCC-1 cells, ZNF671 had a significant effect on both mobility and invasion and may likely affect the expression of target genes involved in these processes. 

### 3.3. Transcriptomic Analysis of ZNF671 Re-Expression Revealed Alterations in Multiple Cancer-Related Signalling Pathways via a Significant Decrease in Oncogenic LINC00665

**Figure 4:**
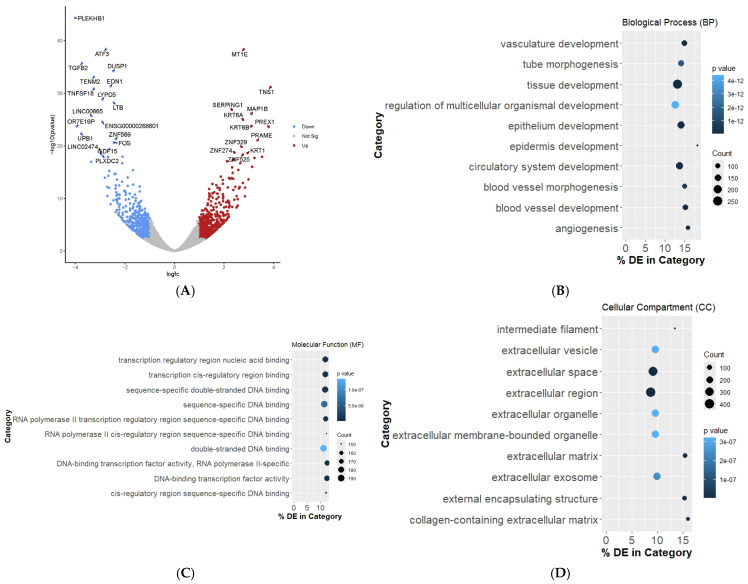
(**A**) Volcano plot of differentially-expressed genes in response to ZNF671 over-expression in UM-SCC-1 oral cancer cells. Down-regulated genes are shown in red. Up-regulated genes are shown in blue. The plot shows the negative log of the *p*-value as a function of the log of the fold-change for each gene. Gene-set enrichment analysis of differentially expressed genes enriched by (**B**) GO:BP (Biological Process) (**C**) GO:MF (Molecular Function) (**D**) GO:CC (Cellular Component). *p*-values for over representation were adjusted for multiple testing using the Benjamini–Hochberg procedure.

In order to obtain a global view of gene expression changes in response to ZNF671 overexpression, we compared transcriptomic profiles of UM-SCC-1 cells overexpressing ZNF671 to those of empty vector control cells using RNA-sequencing technology. This analysis revealed a total of 1729 differentially expressed genes with an adjusted *p*-value of less than 0.05 (Supplementary Table S1). Further restricting our screening of genes to only include those with a log fold change (log_2_FC) of at least 1 (or −1) resulted in 981 differentially expressed genes (540 up-regulated, 441 down-regulated) in response to ZNF671 overexpression (Figure 4A). Most genes identified in both lists of differentially expressed genes were located on chromosome 19, suggesting a role for ZNF671 in the expression of other ZNF proteins.

Gene set enrichment analysis (GSEA) using gene ontology (GO) terms was utilized to group differentially expressed genes along common biological themes (Supplementary Table S2). Most GO:BP categories included those related to genes involved in developmental processes, such as tissue development, epithelium development and development related to vasculature (Figure 4B). GO:MF categories were over-represented by genes associated with transcriptional activity and categories related to regulation of gene expression (Figure 4C). Finally, in terms of cellular components, differentially expressed genes were over-represented in GO categories related to extracellular space and extracellular regions (Figure 4D).

One of the most interesting observations is the dramatic transcription repression of long non-coding RNA LINC00665 and LINC02474 (Figure 4A). We validated the significant decrease in LINC00665 expression in response to ZNF671 expression by real time qPCR in UM-SCC-1 cells (Supplementary Figure S3). We know from previous work that LINC00665 is an oncogenic long non-coding RNA affecting multiple cancer-related signaling pathways via the targeting of tumor suppressive RNAs, such as miR-214-3p and miR424-5p [20]. LINC00665 can participate in the regulation of five signaling pathways to regulate cancer progression, including the Wnt/β-catenin signaling pathway, TGF-β signaling pathway, NF-κB signaling pathway, PI3K/AKT signaling pathway, and MAPK signaling pathway [21]. From these observations, we hypothesize that ZNF671 may represent a novel transcriptional repressor capable of affecting multiple signaling pathways by its targeting of long non-coding RNA LINC00665.

Consistent with the above findings, ZNF671 overexpression resulted in significant decrease in the expression of TGFB2, which plays a significant role in various ongoing cellular mechanisms [22]. Similarly, ZNF671-initiated downregulation of ATF3 is consistent with its role as a negative regulator in the growth and migration of human tongue SCC cells in vitro [23]. Endothelin 1 (EDN1) was also significantly down-regulated in response to ZNF671 overexpression, and it is known to act as a survival factor in oral SCC cells [24]. Other down-regulated genes include EPHB1, ECM1, CCDC34, EMP1 and decorin (DCN), many of which have been shown to have oncogenic properties. A complete list of differentially expressed genes is shown in Supplementary Table S1.

### 3.4. ZNF671 Knockdown in Human Oral Keratinocytes (Hekn) Can Reverse LINC00665 Repression

**Figure 5:**
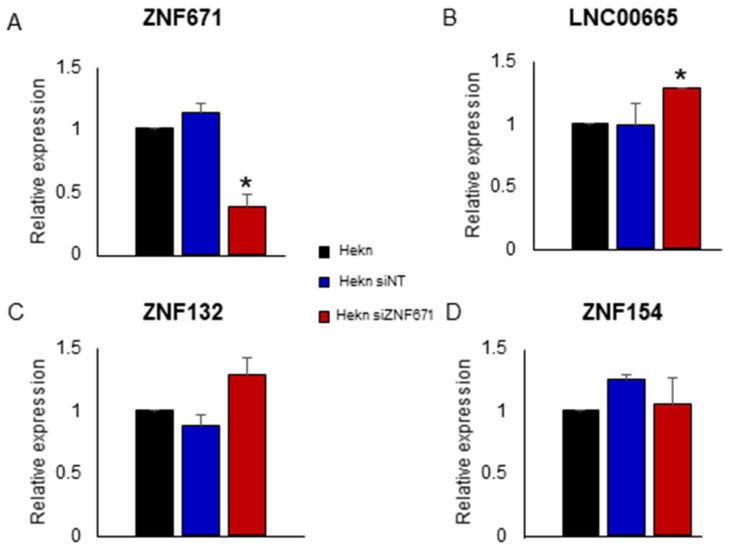
siRNA knockdown of ZNF671 expression in human epithelial keratinocytes (Hekns). Shown are relative expression of (**A**) ZNF671, (**B**) long non-coding RNA LINC00665, (**C**) ZNF132 and (**D**) ZNF154 measured by Taqman qPCR in response to ZNF671 siRNA knockdown (siZNF671). All measurements are compared to parental Hekn cells). Also shown are Hekn cells transfected with a non-targeting control (siNT). All experiments were carried out in duplicate, with duplicate measurements for each experiment. Statistically significant differences between siZNF671 and siNT cells are indicated by an asterisk.

In order to further establish the connection between ZNF671 and oncogenic long non-coding RNA LINC00665, we knocked down ZNF671 expression in human epithelial keratinocytes by siRNA targeting ZNF671 (Figure 5A). Knockdown of ZNF671 expression in these cells was accompanied by a significant increase in LINC00665 expression compared to non-transfected Hekn cells, as measured by real-time qPCR (Figure 5B). This increase was not observed in response to transfection by a non-targeting siRNA control. We also observed that siRNA knockdown of ZNF671 was capable of affecting expression of other ZNF proteins, including ZNF132 on chromosome 19q13 (Figure 5C,D). This is consistent with our findings of a large number of genes located on chromosome 19 among the differentially expressed genes shown in Supplementary Table S1. No change in expression was observed for ZNF154. The exact hierarchy of ZNF protein and ZNF protein targeting and regulation is yet to be determined.

## 4. Discussion

Promoter DNA hypermethylation of tumor suppressor is thought to be one of the most common transcriptional gene silencing mechanisms in human malignancies. In this study, we confirmed that ZNF671 was epigenetically silenced and hypermethylated in a significant number of HNSCC primary tumor samples when compared to adjacent non-tumor samples from the same patient. Our original DNA methylation profiling of head and neck cancers demonstrated that ZNF671 showed elevated DNA hypermethylation as well as reduced gene expression in most head and neck primary tumors [5]. This hypermethylation was observed in all three anatomic subsites of HNSCC (oral cavity, oropharynx and larynx). ZNF671 was also observed in the study by Poage and colleagues, appearing in all three methylation clusters [25].

The epigenetic silencing and tumor suppressive properties of ZNF671 is not confined to these cancers. Recent studies have reported DNA methylation of ZNF671 in cervical cancer [26], nasopharyngeal carcinoma [27], colorectal carcinoma [28] and many others [29]. Results from single cell RNA seq experiments (scRNA) also indicated that ZNF671 inhibited EMT, migration, and invasion of CNS cancers, lung cancer, melanoma, and breast carcinoma in vitro [30]. Overall, elucidating the mechanism of ZNF671 action may provide us with new insights into a role for ZNF671 in cancer treatment.

Survival analysis in two patient cohorts stratifying patients based on ZNF671 expression demonstrated that patients whose primary tumors expressed low levels of ZNF671 showed a significantly decreased survival compared to the rest of the cohort. Cohorts described in this study included head and neck cancers originating from all anatomic subsites of this disease (larynx, hypopharynx, oropharynx and oral cavity). These observations support a role for these proteins in tumor aggressiveness for HNSCC malignancies. The prognostic relevance of ZNF671 was observed not only in HNSCC, but also in breast invasive carcinoma (BRCA), cervical squamous cell carcinoma and endocervical adenocarcinoma (CESC), kidney renal papillary cell carcinoma (KIRP), lung adenocarcinoma (LUAD), pancreatic adenocarcinoma (PAAD), and uterine corpus endometrial carcinoma (UCEC) solid tumors [29].

Little is known about the mechanism of action of ZNF671 in HNSCC, and which genes are the target of its transcriptional suppression. We suspect that ZNF671 represses transcription of key genes involved in cancer progression. Up-regulation of ZNF671 in colorectal carcinoma (CRC) cells resulted in suppressive effects on proliferative ability and metastatic potency via the deactivation of Notch signaling, with decreases in expression of Notch1, NICD, HES1 and HEY1 [28]. Overexpression of ZNF671 in nasopharyngeal cancer (NPC) cells induced S phase arrest by upregulating p21 and downregulating cyclin D1 and c-myc [31]. In urothelial carcinoma (UC) cells, ZNF671 re-expression inhibited tumor growth and invasion, in conjunction with downregulation of cancer stem cell markers (c-KIT, NANOG, OCT4) [32].

Perhaps most interesting in our study is the strong transcriptional repression of oncogenic long non-coding RNA LINC00665 in response to ZNF671 re-expression. LINC00665 is also located on chromosomal 19q13. Recent studies have found that LINC00665 expression was significantly up-regulated in many cancers [21]. LINC00665 plays an oncogenic role in multiple processes, including cancer cell proliferation, migration, and invasion through various molecular mechanisms [20]. These mechanisms often include the sponging of microRNAs but may also involve an encoded biologically active micro-peptide CIP2A-BP. The role of LINC00665 and its micro-peptide CIP2A-BP have yet to be studied in head and neck cancer.

The study as presented does have some limitations. First, only a single HNSCC cell line UM-SCC-1 was utilized in the in vitro studies of ZNF671 overexpression. This represents an oral cavity squamous cell carcinoma cell line isolated from a tumor located on the floor of the mouth of a 73-year-old male patient. This cell line is negative for HPV-16. Moreover, this cell line is derived from a local tumor recurrence and is known to be more invasive in vitro and displays a greater propensity for perineural and lymphatic invasion in vivo. Moreover, the level of ZNF671 protein observed in the overexpression cell line was significantly higher than that which would be observed in vivo and may not be representative of what is seen under normal circumstances. This is common in re-expression studies that express a protein of interest under the control of a strong promoter. To date, our attempts to overexpress ZNF671 in other HNSCC cell lines, including SCC-15 and SCC-25, have not been successful, possibly due to its tumor suppressive properties. We are currently characterizing the ZNF671 overexpression phenotype in the FaDu hypopharyngeal HNSCC cell line. These experiments are now in progress. It is also not clear whether ZNF671 exerts its repressive effect directly on LINC00665 via direct promoter binding, or whether ZNF671 is acting through an as yet unidentified intermediary. Moreover, it is likely that the downregulation of other oncogenic targets listed above may influence the phenotype of ZNF671 overexpressing cells. Experiments to identify direct binding sites by ChIP-seq for ZNF671 in HNSCC cells are now underway.

In conclusion, the work presented here represents a preliminary characterization of a novel tumor suppressor gene (ZNF671) and its role in head and neck carcinogenesis. Future studies by our group are underway to understand the underlying molecular mechanisms regulating ZNF671 expression in HNSCC and other malignancies, and to identify additional downstream genes that are possible targets for its suppression.

## Data Availability

Results of RNA seq analysis are included in Supplementary Table S1.

## Supplementary Materials

The following supporting information can be downloaded at: https://www.mdpi.com/article/10.3390/biomedicines12112482/s1, Figure S1: cBioPortal plot of ZNF671 methylation versus expression; Figure S2: Representative images of Radius cell migration assays; Figure S3: Real-time PCR validation of LINC00665 down-regulation in response to ZNF671 expression; Table S1: Differentially expressed genes in response to ZNF671 overexpression; Table S2: Significantly enriched Gene Ontology categories in response to ZNF671 overexpression.
