# Supplementary material for: ZNF671 Silencing Affects Signaling Pathways in Head and Neck Cancer via Activation of Oncogenic Non-Coding RNAs
Source: Biomedicines. 2024 Oct 29;12(11):2482. doi: 10.3390/biomedicines12112482 (PMC11592198; doi:10.3390/biomedicines12112482)
Supplement: Supplementary file 1 [file biomedicines-12-02482-s001.zip › Supplementary Figures.pdf]

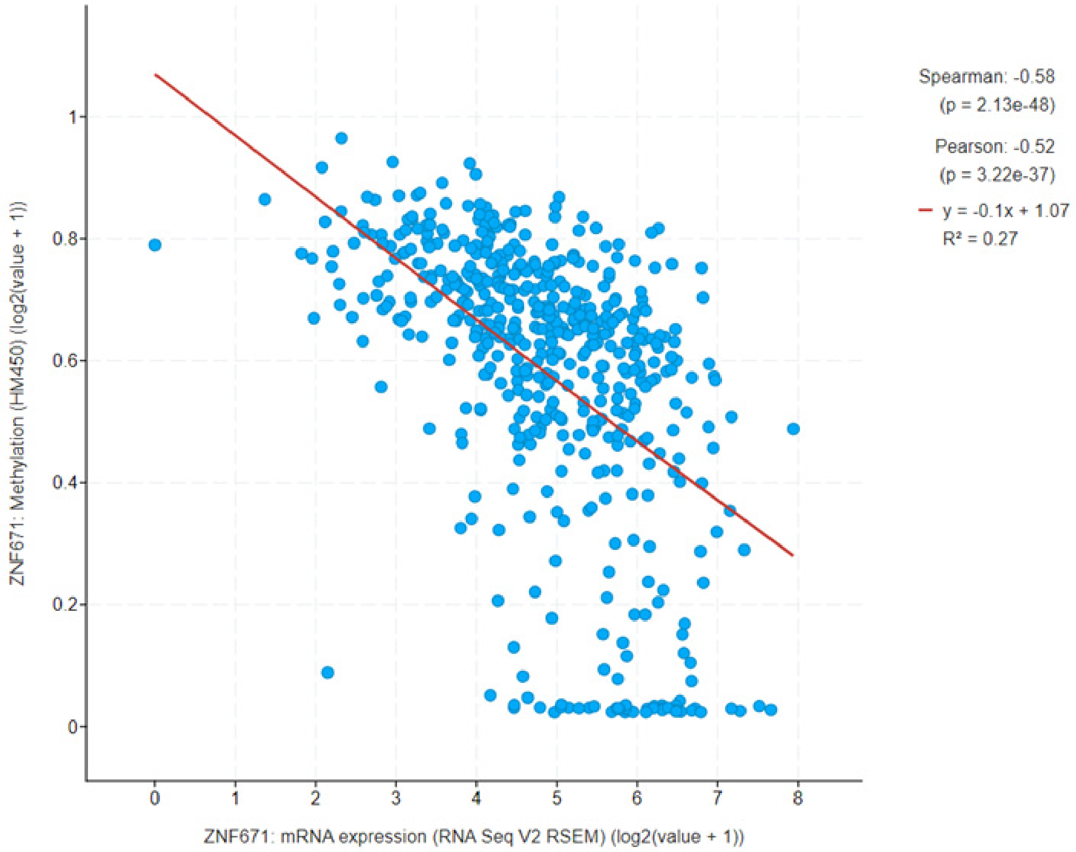

Supplementary Figure S1. cBioPortal plot of ZNF671 promoter methylation (HM450 beadchip beta value) versus ZNF671 mRNA expression (RNA Seq V2 RSEM). Each tumor from the TCGA cohort (n=522) is represented by a blue dot. The red line represents a linear regression line of best fit through the data (https://www.cbioportal.org/).

UM-SCC-1 cells

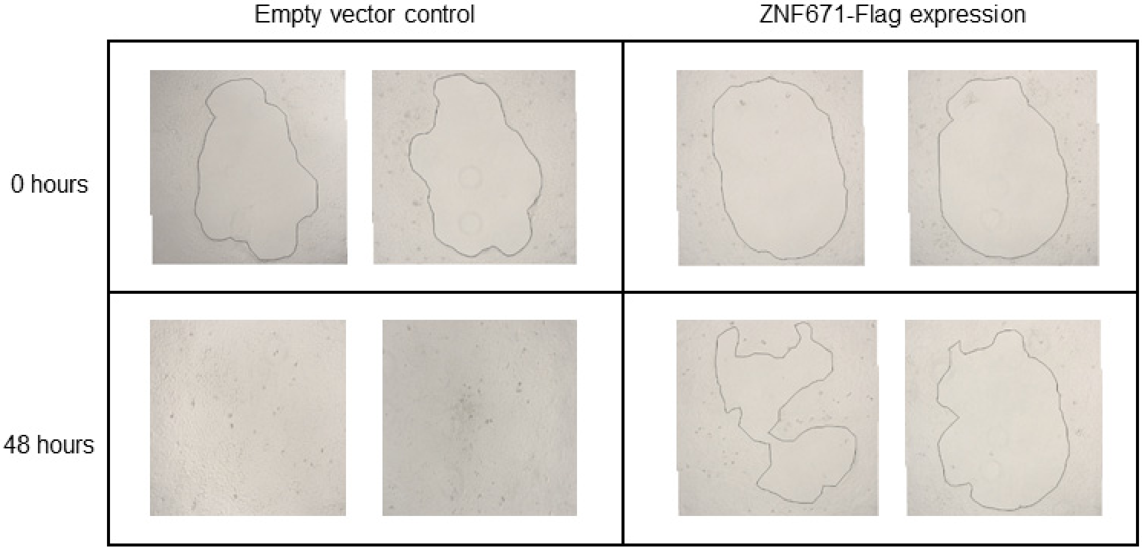

Supplementary Figure S2. Representative images of duplicate Radius cell migration assays comparing migration of HNSCC cancer UM-SCC-1 cells containing empty vector or ZNF671-Flag construct. Images were taken at 0 and 48 hours post gel removal.

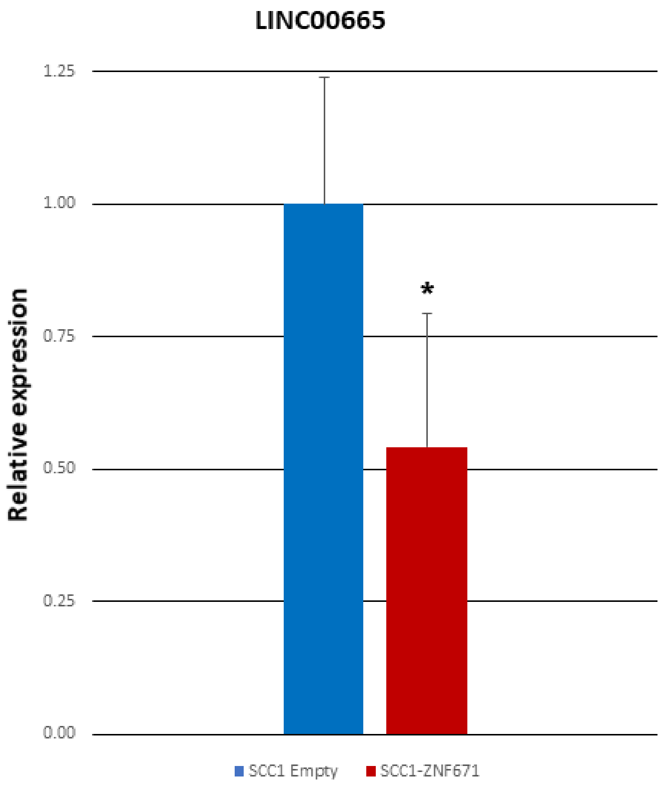

Supplementary Figure S3. Relative expression of long non-coding RNA LINC00665, as measured by Taqman qPCR, in response to ZNF671 overexpression in UM-SCC-1 cancer cells (red). Measurements are relative to the UM-SCC-1 empty vector control cells (blue). All experiments were carried out in duplicate, with duplicate measurements for each experiment. Statistically significant differences between ZNF671-overexpressing and empty vector control cells are indicated by an asterisk.
